# Murid herpesvirus-4 lacking thymidine kinase reveals route-dependent requirements for host colonization

**DOI:** 10.1099/vir.0.010603-0

**Published:** 2009-06

**Authors:** Michael B. Gill, Debbie E. Wright, Christopher M. Smith, Janet S. May, Philip G. Stevenson

**Affiliations:** Division of Virology, Department of Pathology, University of Cambridge, Cambridge, UK

## Abstract

Gammaherpesviruses infect at least 90 % of the world's population. Infection control is difficult, in part because some fundamental features of host colonization remain unknown, for example whether normal latency establishment requires viral lytic functions. Since human gammaherpesviruses have narrow species tropisms, answering such questions requires animal models. Murid herpesvirus-4 (MuHV-4) provides one of the most tractable. MuHV-4 genomes delivered to the lung or peritoneum persist without lytic replication. However, they fail to disseminate systemically, suggesting that the outcome is inoculation route-dependent. After upper respiratory tract inoculation, MuHV-4 infects mice without involving the lungs or peritoneum. We examined whether host entry by this less invasive route requires the viral thymidine kinase (TK), a gene classically essential for lytic replication in terminally differentiated cells. MuHV-4 TK knockouts delivered to the lung or peritoneum were attenuated but still reached lymphoid tissue. In contrast, TK knockouts delivered to the upper respiratory tract largely failed to establish a detectable infection. Therefore TK, and by implication lytic replication, is required for MuHV-4 to establish a significant infection by a non-invasive route.

## INTRODUCTION

Gammaherpesviruses persist in lymphocytes and use lymphocyte proliferation to amplify their latent genome loads. Viral immune evasion (Stevenson, 2004) makes this process hard to stop. It also makes long-term infection hard to clear. The initial establishment of lymphocyte infection by virions entering a naive host is therefore a key target for infection control.

Gammaherpesviruses, like many other mammalian herpesviruses, are thought to be transmitted via saliva (Yao *et al.*, 1985). Epstein–Barr virus (EBV), as the most intensively studied example, provides something of an archetype. It infects epithelial cells poorly *in vitro*, and has therefore been proposed to infect *in vivo* by saliva reaching B cells in tonsillar crypts (Faulkner *et al.*, 2000; Thorley-Lawson, 2001), where infection is abundant during infectious mononucleosis. However, infectious mononucleosis occurs at least 1 month after actual transmission (Hoagland, 1964). Therefore, tonsillar infection could just as easily represent EBV exit. One prediction of the direct B cell infection hypothesis, that antibodies to gp350, which block B cell infection *in vitro* (Thorley-Lawson & Geilinger, 1980), would reduce host entry *in vivo*, was not fulfilled (Sokal *et al.*, 2007). Blocking gp350-independent (non-B cell) EBV infection (Janz *et al.*, 2000) would require a different vaccine. Therefore it is important for infection control to understand how gammaherpesviruses enter their hosts.

Both EBV and the other known human gammaherpesvirus, the Kaposi's sarcoma-associated herpesvirus (KSHV), are difficult to study *in vivo*. Therefore the small animal model provided by murid herpesvirus-4 (MuHV-4) (Nash *et al.*, 2001; Stevenson & Efstathiou, 2005) provides a useful means of analysing gammaherpesvirus pathogenesis. MuHV-4 is more closely related to KSHV than to EBV, but epithelial and B cell tropisms are common to all three viruses, and presumably pre-date their divergence; similar routes of host colonization are therefore likely. The main natural host of MuHV-4 appears to be yellow-necked mice (*Apodemus flavicollis*) (Kozuch *et al.*, 1993). However, it behaves like a natural parasite also in *Mus musculus*-derived laboratory strains, persisting without disease unless there is immunosuppression (Virgin & Speck, 1999). Moreover its immune-evasion genes, typically the most host-restricted of viral functions, seem to work normally in laboratory mice, even showing strain specificity (Boname *et al.*, 2004). MuHV-4 infection of laboratory mice therefore seems to offer a reasonable physiological model of gammaherpesvirus infection.

In the standard intranasal MuHV-4 infection model, deep general anaesthesia and a large inoculum volume are used to deliver virions directly to the lung alveoli, where lytic replication occurs. Vaccination against viral lytic antigens largely blocks this replication, but does not block latency establishment (Stevenson *et al.*, 1999). Thymidine kinase (TK)-deficient mutants show a severe lytic replication defect in lungs, but still establish normal long-term latency in spleens (Coleman *et al.*, 2003). The establishment of B-cell latency after lung infection is therefore independent of the extent of viral lytic replication. Completely replication-deficient MuHV-4 genomes can also persist after delivery to the lung alveoli or the peritoneal cavity. Thus, ORF6^−^ MuHV-4 infects peritoneal macrophages, but not splenic B cells after intraperitoneal inoculation (Tibbetts *et al.*, 2006); ORF31^−^ MuHV-4 reaches the spleen after intraperitoneal inoculation, but not after lung inoculation (Kayhan *et al.*, 2007); and ORF50^−^ MuHV-4 colonizes B cells in the lung, but not in the spleen, after lung inoculation (Moser *et al.*, 2006). This last result is hard to understand, because B cells infected in the lung should recirculate to the spleen, and it is possible that some of the PCR-based assays of host colonization used with replication-deficient mutants detected input viral debris rather than bona fide infected cells. The conservative conclusion would be that systemic lymphoid tissue is not readily accessible to MuHV-4 delivered by the intranasal route. Nevertheless, all these studies concluded that replication-deficient viruses established significant persistent infections.

The value of an animal model depends not just on sophisticated genetic techniques, but on ecological considerations such as how virions might normally enter their hosts. Intranasal virus inoculation is more likely than intraperitoneal inoculation to reflect natural infection. However, direct virus delivery to the lung may also be misleading. MuHV-4 given intranasally in low volumes without anaesthesia replicates lytically just in the nose (Milho *et al.*, 2009). A persistent, systemic infection is nevertheless robustly established even with low-dose inoculations. In contrast, orally delivered virus is very poorly infectious (Milho *et al.*, 2009). Therefore the nose, but not the lung, is a plausible natural portal of host entry.

Distinguishing upper and lower respiratory tract entry routes is important, as they involve very different epithelial surfaces. In order to explore further the requirements of MuHV-4 for host colonization via the upper respiratory tract, we infected mice with TK-deficient mutants (Coleman *et al.*, 2003). We used these in preference to completely replication-deficient mutants for two reasons: first, we are interested in viral functions that are specifically required for replication *in vivo*, and TK^−^ MuHV-4 replicates normally *in vitro*; second, the normal *in vitro* replication of TK^−^ mutants places less reliance on measuring host colonization by viral DNA loads, which do not distinguish viable genomes from non-infectious debris. In contrast to lung or peritoneal infections, TK^−^ virions delivered to the upper respiratory tract failed to establish a detectable infection. The herpes simplex virus (HSV) TK is largely redundant for epithelial infection, but is essential for lytic replication in terminally differentiated neurons (Efstathiou *et al.*, 1989; Coen *et al.*, 1989; Valyi-Nagy *et al.*, 1994). Therefore, MuHV-4 may have to pass through terminally differentiated cells when infecting via the nose, suggesting that TK-based therapies have the potential to block gammaherpesvirus spread.

## METHODS

### Mice.

Female BALB/c mice were infected with MuHV-4 when 6–12 weeks old. Intranasal infections were either in 30 μl under deep general anaesthesia (lower respiratory tract infection) or in 5 μl under light anaesthesia (upper respiratory tract infection). Intraperitoneal infections were in 300 μl. For luciferase imaging, mice were given intraperitoneal injections of luciferin (2 mg per mouse), anaesthetized with isoflurane, then scanned with an IVIS Lumina (Caliper Life Sciences). Quantitative comparisons used the maximum radiance (photons s^−1^ cm^−2^ steradian^−1^) over each region of interest, relative to a negative control region. All experiments conformed to local animal ethics regulations and Home Office Project Licence 80/1992.

### Cells.

BHK-21, 293T, NIH-3T3-CRE (Stevenson *et al.*, 2002) and RAW-264 cells were propagated in Dulbecco's modified Eagle's medium (Invitrogen), supplemented with 2 mM glutamine, 100 U penicillin ml^−1^, 100 *μ*g streptomycin ml^−1^ and 10 % fetal calf serum. Peritoneal cells were obtained by a post-mortem injection and aspiration of 10 ml PBS. Typically 50 % of the cells recovered were macrophages (CD19^−^F4/80^+^ by flow cytometry). The remainder were B cells (CD19^+^F4/80^−^). Selection by adherence to plastic increased the proportion of macrophages to >90 %.

### Viruses.

The TK^−^KAN^−^ mutant, its revertant, and the TK^−^DEL mutant have been described previously (Coleman *et al.*, 2003). We generated LUC^+^ TK knockouts by shuttling the M3-LUC reporter construct (Milho *et al.*, 2009) into the TK^−^KAN^−^ bacterial artificial chromosome (BAC). We also combined the TK^−^KAN^−^ mutation with enhanced GFP (eGFP)-tagged gM (Smith *et al.*, 2007) and with gp150 disruption (M7^−^STOP) (de Lima *et al.*, 2004). The integrity of each recombinant BAC was confirmed by restriction endonuclease mapping. Infectious virions were recovered by transfecting BAC DNA into BHK-21 cells. For *in vivo* experiments, the loxP-flanked viral BAC/eGFP cassette was removed by passage through NIH-3T3-CRE cells. Virus stocks were grown in BHK-21 cells (de Lima *et al.*, 2004).

### Infectivity assays.

Virus stocks were titrated by plaque assay on BHK-21 cells (de Lima *et al.*, 2004): cell monolayers were incubated with virus (2 h, 37 °C), overlaid with 0.3 % carboxymethylcellulose, and 4 days later fixed and stained for plaque counting. Infectious virus in *ex vivo* tissues was measured by plaque assay of freeze–thawed tissue homogenates. To titre noses, we removed a block of tissue bounded by (i.e. not including) anteriorly the cartilaginous tip of the nose, posteriorly the orbits, laterally the zygomatic arches, ventrally the palate and dorsally the nasal bones. This region contained all the luciferase signal measureable by *ex vivo* charge-coupled device (CCD) camera scanning. Bone fragments were discarded after homogenization. The latent virus in *ex vivo* tissues was measured by infectious centre assay (de Lima *et al.*, 2004): spleen or superficial cervical lymph node (SCLN) suspensions were co-cultured with BHK-21 cells, then fixed and stained for plaque counting after 4 days. Pre-formed infectious virus titres in lymphoid tissue, as measured by plaque assay of freeze–thawed cells, were always <1 % of infectious centre assay titres.

### Viral genome quantification.

Viral genome loads were measured by real-time PCR (Gaspar *et al.*, 2008). DNA (50–80 ng) was extracted from *ex vivo* organs (Wizard genomic DNA purification kit; Promega) and used to amplify MuHV-4 genomic co-ordinates 4166–4252 (Rotor Gene 3000; Corbett Research). The PCR products were quantified by hybridization with a Taqman probe (genomic coordinates 4218–4189) and converted to genome copies by comparison with a standard curve of cloned plasmid template, amplified in parallel. Cellular DNA was quantified in parallel by amplifying part of the adenosine phosphoribosyltransferase gene (forward primer 5′-GGGGCAAAACCAAAAAAGGA-3′, reverse primer 5′-TGTGTGTGGGGCCTGAGTC-3′, probe 5′-TGCCTAAACACAAGCATCCCTACCTCAA-3′).

### ELISA.

MuHV-4 virions were recovered from infected BHK-21 cell supernatants by ultracentrifugation, disrupted with 0.05 % Triton X-100 in 50 mM sodium carbonate buffer (pH 8.5), and coated (18 h, 4 °C) onto Maxisorp ELISA plates (Nalgene Nunc). The plates were washed three times in PBS/0.1 % Tween-20, blocked with 2 % BSA in PBS/0.1 % Tween-20 (1 h, 23 °C), then incubated with threefold serum dilutions from MuHV-4-exposed mice (1 h, 23 °C). The plates were then washed four times in PBS/0.1 % Tween-20, incubated (1 h, 23 °C) with alkaline phosphatase-conjugated goat anti-mouse IgG-Fc pAb (Sigma), washed five times, and developed with nitrophenylphosphate (Sigma). Absorbance was read at 405 nm (Bio-Rad Benchmark ELISA plate reader).

## RESULTS

### Total respiratory tract infection by TK^−^ MuHV-4

MuHV-4 TK knockouts have a severe lytic replication defect in lungs as measured by plaque assay (Coleman *et al.*, 2003). Viral luciferase expression provides an alternative means of tracking infection that can be applied to live mice (Milho *et al.*, 2009). We expressed luciferase from an early lytic MuHV-4 promoter, making it a subtly different readout to plaque assays, which also require viral late functions to form infectious virions. Since TK is thought to function mainly in viral DNA replication, luciferase expression should provide a more sensitive measure of host colonization by TK^−^ mutants. Like other TK knockouts (Coleman *et al.*, 2003), luciferase^+^TK^−^ MuHV-4 replicated normally *in vitro*. Its TK status was confirmed by staining infected cells with the TK-specific mAb CS-4A5 (May *et al.*, 2008) (data not shown).

**Fig. 1. f1:**
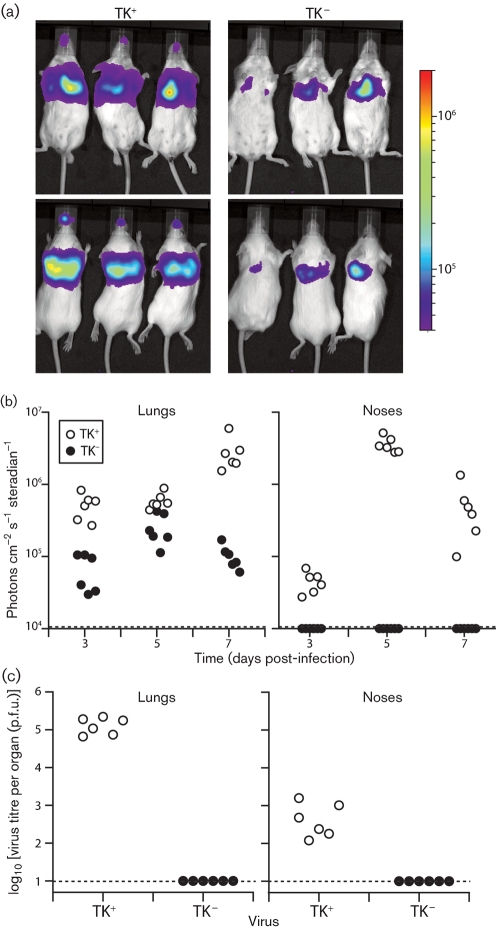
Luciferase imaging of TK^−^ MuHV-4 infection in the lower respiratory tract. (a) Mice were infected intranasally (10^3^ p.f.u., 30 μl) under general anaesthesia with TK^+^ or TK^−^ luciferase^+^ MuHV-4, then after 3 days received injections of luciferase and were imaged by CCD camera scanning. The images show dorsal and ventral views of three mice per group. The bar shows the colour scheme of relative signal intensity. (b) Maximum luciferase signal intensities in the noses and lungs of mice (six per group) infected with TK^+^ or TK^−^ luciferase^+^ MuHV-4 were monitored as in (a). Each point shows the signal of one mouse. The dashed lines show lower limits of detectable signal intensity. (c) Plaque assays of mouse noses and lungs, 7 days after infection with TK^+^ or TK^−^ luciferase^+^ MuHV-4 as in (a). Each point shows the titre of one mouse. The dashed lines show lower limits of assay sensitivity.

After intranasal virus inoculation (10^3^ p.f.u.) under deep anaesthesia (Fig. 1), luciferase expression was detectable in the lungs of mice given either TK^+^ or TK^−^ viruses. However, the TK^−^ mutant showed no luciferase signal in the nose (Fig. 1a, b[Fig f1]). Therefore the TK-dependent reduction in early lytic gene expression, presumably reflecting reduced lytic spread, was greater in noses than in lungs. Infectious TK^−^ MuHV-4 was undetectable in both noses and lungs by plaque assay (Fig. 1c[Fig f1]).

### Upper respiratory tract infection by TK^−^ MuHV-4

**Fig. 2. f2:**
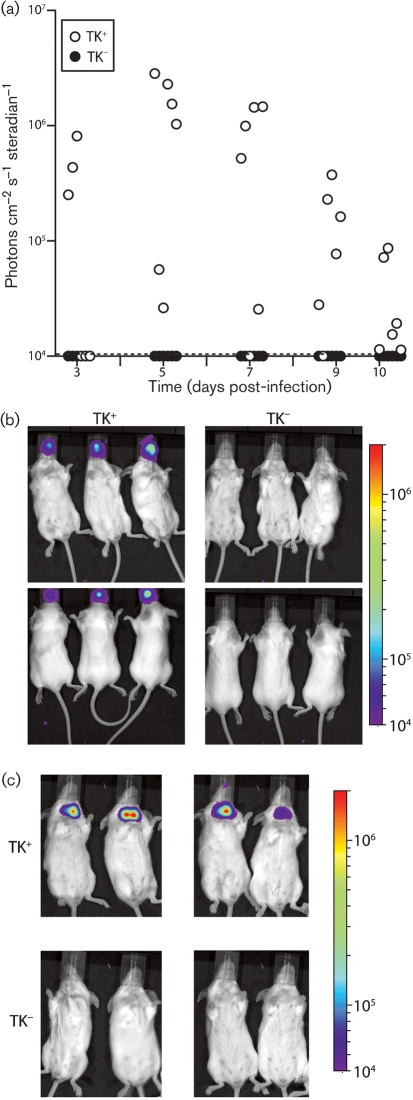
TK^−^ MuHV-4 infection of the upper respiratory tract. (a) Mice were inoculated intranasally with TK^+^ or TK^−^ luciferase^+^ MuHV-4 (10^3^ p.f.u. in 5 μl), then imaged for luciferase expression over the next 10 days. Each point shows the maximum radiance signal of one mouse. The dashed line shows the lower limit of assay sensitivity. (b) Example dorsal and ventral views of luciferase signals 3 days after infection with 5 μl TK^+^ or TK^−^ luciferase^+^ MuHV-4. (c) Luciferase signals at 10 days after infection of four mice each with 5 μl TK^+^ or TK^−^ luciferase^+^ MuHV-4. The neck signal is from the superficial cervical lymph nodes.

We explored the TK-dependent upper respiratory tract infection defect further by low-volume intranasal inoculation (Fig. 2). This delivers virus more accurately to the nose, because none of the inoculum is aspirated into the lung. Again there was no evidence of luciferase expression in the nose by the TK knockout (Fig. 2a, b[Fig f2]). Nor was there a later signal in the neck (Fig. 2c[Fig f2]), which corresponds to viral latency establishment in the SCLN (Milho *et al.*, 2009). Therefore, TK^−^ MuHV-4 appeared not to establish a significant infection via the upper respiratory tract.

**Fig. 3. f3:**
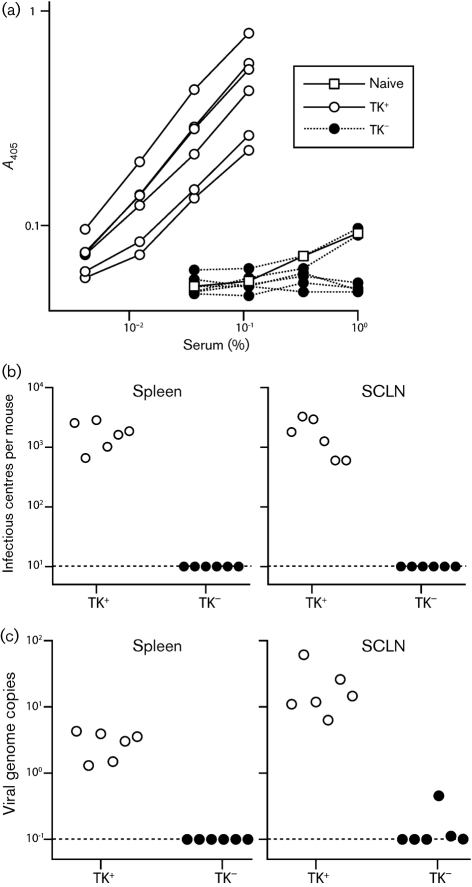
No late colonization of mice by TK^−^ MuHV-4 delivered to the upper respiratory tract. (a) Mice were inoculated intranasally (10^3^ p.f.u., 5 μl) with TK^+^ or TK^−^ luciferase^+^ MuHV-4, then analysed for MuHV-4-specific IgG at 1 month post-infection by ELISA. Each line shows the result for one mouse. Pooled naive sera provide the negative control. (b) The same mice as in (a) were analysed for reactivatable splenic virus by infectious centre assay. Each point shows the titre for one mouse. The dashed lines show lower limits of assay sensitivity. (c) The same samples as in (b) were analysed for viral genomes by real-time PCR of DNA from spleens or SCLN. Each point shows the viral genome copy number of one mouse, normalized by the host genome copy number of the same sample (1000× viral genomes per host genome). The dashed lines show lower limits of assay sensitivity.

TK^−^ MuHV-4 delivered to the lung establishes latency despite its lytic replication defect: reactivatable virus is present in the spleen and antibodies to viral lytic antigens are present in serum by 1 month post-inoculation (Coleman *et al.*, 2003). Given the minimal primary lytic spread of TK^−^ mutants, this antibody response presumably reflects latency-associated lymphoproliferation, followed by reactivation. At any rate, it provides a sensitive measure of host colonization. TK^−^ MuHV-4 delivered to the upper respiratory tract had elicited no detectable antibody response after 1 month (Fig. 3a[Fig f3]). Infectious centres were also undetectable in spleens or SCLN (Fig. 3b[Fig f3]), and only one mouse showed a viral genome PCR signal above background levels (Fig. 3c[Fig f3]). Even that signal was very low, suggesting sample contamination or the detection of input viral debris transported to draining lymph nodes. Splenic PCR signals, which are probably a more stringent test of significant host colonization, were all negative. Therefore, TK^−^ MuHV-4 seemed to infect mice poorly via the upper respiratory tract.

**Fig. 4. f4:**
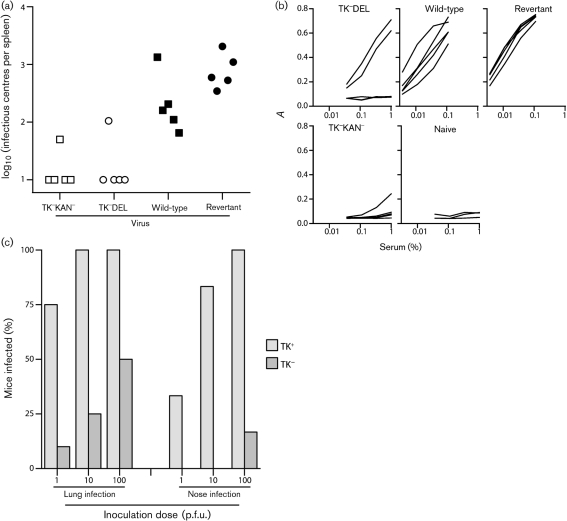
Analysis of infection by luciferase^−^ TK mutants. (a) Mice were inoculated intranasally (10^4^ p.f.u., 5 μl) with TK^+^ (wild-type or revertant) or TK^−^ viruses (TK^−^KAN^−^, TK^−^DEL). One month later spleens were assayed for replication-competent virus by infectious centre assay. (b) Sera from the same mice were assayed for MuHV-4-specific IgG by ELISA. Sera from three age-matched naive mice were assayed in parallel. Each line shows the result for one mouse. (c) Mice were inoculated intranasally (10^3^ p.f.u.) with TK^+^ or TK^−^ MuHV-4 in 30 μl (lung infection) or 5 μl (nose infection), and 1 month later assayed for MuHV-4-specific serum IgG as in (b). Each mouse was scored as infected or not depending on whether the absorbance value with a 1 : 100 serum dilution exceeded that of age-matched naive controls. Each bar shows the percentage positive of 6–10 mice per group. Except for the 1 p.f.u. nose and 100 p.f.u. lung infections, all TK^−^ infection rates were significantly less than those for TK^+^ (*P*<0.02 by Fisher's exact test).

We then tested our original, luciferase^−^ TK knockouts (TK^−^KAN^−^, TK^−^DEL) for host colonization via the upper respiratory tract (Fig. 4). We also increased the inoculation dose to 10^4^ p.f.u. so as to test maximally any resistance to infection. This confirmed the finding with the luciferase^+^ TK mutant: only two out of ten mice exposed to high dose TK^−^ MuHV-4 had detectable virus by infectious centre assay of spleens (Fig. 4a[Fig f4]), and only three out of ten had detectable antibody responses (Fig. 4b[Fig f4]), whereas all control mice were infected. As 10 p.f.u. wild-type MuHV-4 readily infects mice via the upper respiratory tract (Milho *et al.*, 2009), TK knockouts were at least 100-fold less capable of establishing a detectable infection. Comparing different doses of TK^+^ and TK^−^ viruses (Fig. 4c[Fig f4]), and assaying infection by ELISA of serum for MuHV-4-specific IgG at 1 month post-infection (the most sensitive measure), showed TK^−^ infection to be inefficient via either the upper or the lower respiratory tract. Upper respiratory tract TK^−^ infection appeared to be less efficient, but the difference between inoculation routes did not reach statistical significance.

### Peritoneal infection by TK^−^ MuHV-4

**Fig. 5. f5:**
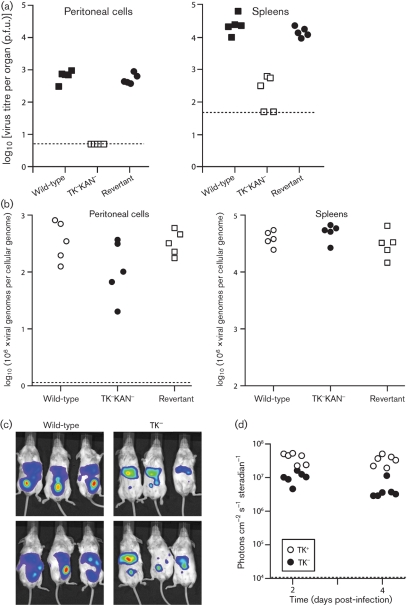
Intraperitoneal infection by TK^−^ MuHV-4. (a) Mice were infected intraperitoneally (10^3^ p.f.u.) with TK^+^ (WT, REV) or TK^−^ (TK^−^KAN^−^) MuHV-4. Macrophages were then recovered by peritoneal lavage, and these and spleen cells were titrated for recoverable virus by infectious centre assay. Dashed lines show the lower limits of assay sensitivity. Each point shows the titre for one mouse. (b) Viral genome loads in DNA from the peritoneal and spleen cell samples in (a) were quantified by real-time PCR. Each point shows the value for one mouse, normalized by the host genome copy number of the same sample. The dashed line shows the lower limit of signal detection. (c) Mice were infected intraperitoneally (10^3^ p.f.u.) with TK^+^ or TK^−^ luciferase^+^ MuHV-4, and 2 days later imaged by luciferin injection and CCD camera scanning. The colour scheme of relative signal intensity is as for Figs 1 and 2. (d) Maximum radiance values after TK^−^ and TK^+^ intraperitoneal virus inoculations were calculated from scanned images using Living Image software. Each point shows the value for one mouse. The dashed line shows the lower limit of signal detection.

Several studies of host colonization by replication-deficient MuHV-4 mutants have used intraperitoneal inoculation. For the purpose of comparison, therefore, we also looked at this route of infection with TK^−^ mutants (Fig. 5). We first tested our published TK^−^KAN^−^ virus by the standard infectious centre assay of spleens and peritoneal macrophages (Fig. 5a[Fig f5]). Some infectivity was recoverable from spleens, although at least tenfold less than with TK^+^ controls. No TK^−^ infectivity was recovered from peritoneal macrophages. Real-time PCR showed relatively normal TK^−^ viral genome loads in peritoneal cells and spleens (Fig. 5b[Fig f5]). Therefore, the low plaque titres of the TK^−^KAN^−^ mutant appeared to reflect mainly poor infectious virion production. Live imaging of infected cells (Fig. 5c, d[Fig f5]) supported this idea, with relatively little TK-dependent deficit.

**Fig. 6. f6:**
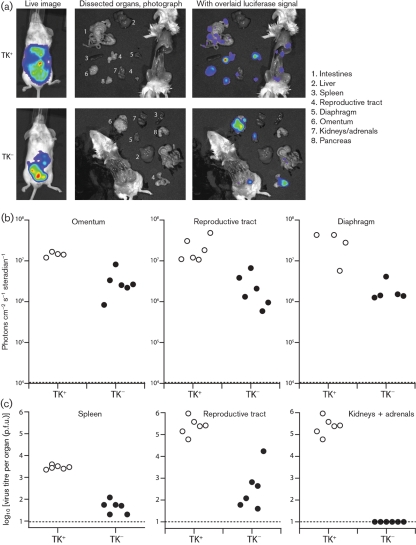
Intraperitoneal infection by TK^−^ MuHV-4: individual organs. (a) Mice were infected intraperitoneally with TK^+^ or TK^−^ luciferase^+^ MuHV-4 (10^3^ p.f.u.), and 5 days later imaged for luciferase expression, first live and then post-mortem after dissection. Representative examples are shown. (b) Quantification of data equivalent to those of (a), showing maximum radiance values for the omentum, reproductive tract and diaphragm of six mice per group. Each point shows the signal for one mouse. The dashed lines show signal detection limits. (c) Virus titres of samples from equivalent infections to (b), measured by plaque assay. Each point shows the titre for one mouse. The dashed lines show lower limits of detection.

We defined the sources of luciferase signals by *ex vivo* imaging of dissected organs (Fig. 6). TK^+^ MuHV-4 showed widespread peritoneal infection, with luciferase expression in intestines, spleen, reproductive tract, diaphragm, adrenals and pancreas (Fig. 6a[Fig f6]). The apparent lack of kidney, liver or spleen signals in Fig. 6(a) reflects only that the image sensitivity was set low to keep the strong reproductive tract signal on scale – none of these organs was completely luciferase-negative. Intraperitoneal TK^−^ luciferase signals were generally lower than wild-type, but again no peritoneal organs were consistently luciferase-negative. Therefore, the pattern was very different from that for upper respiratory tract infection, where the noses of mice given intranasal TK^−^ MuHV-4 were always luciferase-negative. The strongest and most consistent intraperitoneal TK^−^ luciferase signals, as with TK^+^, were those of the reproductive tract, omentum and diaphragm. Fig. 6(b) shows quantitative comparisons of four to six mice per group.

Infectious virus (TK^+^ or TK^−^) was hard to recover from the omentum because the associated fat was toxic to BHK-21 cells, and infectious virus was recovered only inconsistently from the diaphragm. The reproductive tract was the most reliable source of both TK^+^ and TK^−^ infectious virions. The spleen also yielded infectious TK^−^ virions, as before (Fig. 5a[Fig f5]). Both organs showed substantially greater TK-dependent virion production defects than luciferase expression defects. The kidneys and adrenals were positive only for TK^+^ virions. Nevertheless, TK^−^ mutants remained infectious by the intraperitoneal route.

### Macrophage infection by TK^−^ MuHV-4

**Fig. 7. f7:**
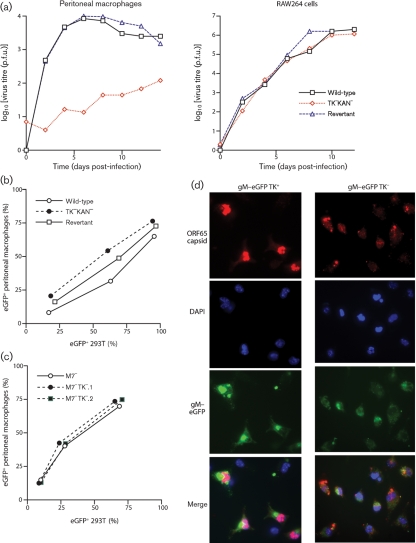
Macrophage infection by TK^+^ and TK^−^ MuHV-4. (a) Peritoneal macrophages from naive mice or RAW-264 cells were infected (0.01 p.f.u. per cell, 2 h) with TK^+^ (wild-type or revertant) or TK^−^ viruses, then washed twice in PBS and cultured at 37 °C. Samples were titrated for infectious virus by plaque assay at the times shown. (b) 293T epithelial cells or peritoneal macrophages were exposed to eGFP^+^ TK^+^ or TK^−^ viruses at different multiplicities, then 24 h later assayed for eGFP expression by flow cytometry. Since MuHV-4 infects 293T cells better than peritoneal macrophages, the infecting doses for the latter were 10 times higher for each virus. The peritoneal macrophages were treated with LPS (100 ng ml^−1^, 6 h) before assay to maximize HCMV IE1 promoter-driven viral eGFP expression. (c) As in (b), we compared TK^+^ and TK^−^ viruses for their capacity to infect either 293T cells or peritoneal macrophages, except that all viruses were additionally disrupted for M7 (gp150). This allowed us to use equivalent virus doses for each cell type, as M7^−^ MuHV-4 infects macrophages more efficiently than the wild-type. (d) Peritoneal macrophages were exposed (2 p.f.u. per cell) to TK^+^ or TK^−^ viruses carrying eGFP-tagged gM (green). The cells were fixed 18 h later, permeabilized and stained for the ORF65 capsid component (red). Nuclei were counterstained with DAPI (blue).

Since peritoneal macrophages showed no sign of producing infectious TK^−^ virions, we used them to define further the nature of the TK-dependent infection defect (Fig. 7). Growth curves showed poor TK^−^ virion production by primary peritoneal macrophages, but not by the transformed monocyte/macrophage cell line RAW-264 (Fig. 7a[Fig f7]). TK knockouts also failed to replicate in bone-marrow-derived dendritic cells (data not shown). TK^−^ virion entry into peritoneal macrophages did not seem to be impaired, as, for a given level of viral eGFP expression in 293T cells, eGFP expression in peritoneal macrophages was similar between TK^−^ and TK^+^ viruses (Fig. 7b[Fig f7]). We have shown previously that this viral eGFP expression is independent of lytic replication (Smith *et al.*, 2007).

MuHV-4 typically infects macrophages tenfold less efficiently than it infects fibroblasts, presumably because macrophages express only low levels of the heparan sulfate that MuHV-4 requires for cell binding (Gillet *et al.*, 2007). Therefore, it was possible that a general inefficiency of entry masked an additional TK-dependent defect. We addressed this by disrupting the MuHV-4 M7 ORF, which encodes gp150. gp150^−^ virions are heparan sulfate-independent and consequently infect macrophages as efficiently as they infect fibroblasts (de Lima *et al.*, 2004). Viral eGFP expression in peritoneal macrophages on an M7^−^ virus background remained TK-independent (Fig. 7c[Fig f7]).

Immune fluorescence of *ex vivo* peritoneal macrophage infections (Fig. 7d[Fig f7]) showed that TK^+^ and TK^−^ virions were both endocytosed (cytoplasmic glycoprotein and capsid staining), but that only TK^+^ virions gave new late lytic gene expression (nuclear capsid staining). This result was consistent with TK^−^ MuHV-4 infecting but then failing to replicate in peritoneal macrophages *in vivo* (Fig. 5). Therefore, the TK-dependent macrophage infection defect matched the pattern expected for a virus not producing enough phosphorylated thymidine to replicate in terminally differentiated cells.

## DISCUSSION

Our aim in analysing gammaherpesvirus host entry has been to reveal new targets for infection control. We showed here that MuHV-4 requires its TK to establish a significant infection of mice via the upper respiratory tract. More invasive infections were less TK-dependent, but they are less physiological. HSV requires its TK to replicate in terminally differentiated neurons. Gammaherpesvirus TKs are not necessarily the same, but TK^−^ MuHV-4 showed a macrophage infection defect consistent with impaired viral DNA replication in terminally differentiated cells. A similar requirement for host colonization by human gammaherpesviruses would make their lytic genes potential targets for infection control.

The phenotype of TK^−^ MuHV-4 raised some general questions about gammaherpesvirus host entry. We assume that TK^−^ virions still infected cells in the upper respiratory tract, but that the number of cells supporting early lytic gene expression was insufficient for a detectable luciferase signal and without TK, infection did not spread. This would make B cells an unlikely primary target for incoming virions, as TK^−^ mutants can both replicate in B cells and drive their proliferation (Coleman *et al.*, 2003). The poor *in vitro* binding of cell-free MuHV-4 virions to B cells (Gillet & Stevenson, 2007) would also argue against direct infection *in vivo*. The poor replication of TK^−^ MuHV-4 in macrophages could explain TK-dependent host entry if macrophages were a significant primary target. However, cell-free virions also infect macrophages rather poorly (Rosa *et al.*, 2007), and it is unclear what proportion of nasal antigens are sampled by macrophages or dendritic cells. Macrophages are a more plausible primary target in lung alveoli and in the peritoneal cavity, where they have an important scavenging function, but infection in these sites must be considered atypical.

Epithelial cells remain a plausible primary target. However, any epithelial entry model must explain how MuHV-4 virions, which are highly heparan sulfate-dependent *in vitro*, cope with epithelial heparan sulfate being predominantly basolateral *in vivo* (Hayashi *et al.*, 1987). The failure of wild-type MuHV-4 to infect mice orally (Milho *et al.*, 2009) also argues for a more anatomically restricted target. Therefore epithelial entry is far from certain. One possibility suggested by the phenotype of TK^−^ HSV (Efstathiou *et al.*, 1989; Coen *et al.*, 1989; Valyi-Nagy *et al.*, 1994) is that incoming MuHV-4 infects olfactory neurons. These display heparan sulfate on syndecan-3 (Raulo *et al.*, 1994) and engage in environmental sampling. However, TK might be also required simply because the accessible epithelial cells are terminally differentiated. While TK^−^ HSV shows only a mild epithelial replication defect, experimental HSV infection involves deliberate scarification that would stimulate local cell division; the normal state of most cells is quiescence. Notably, we recovered infectious TK^−^ virions from only the reproductive tract and spleen, where mitotic rates are relatively high. Therefore further analysis is required to identify precisely where in the upper respiratory tract host colonization by TK^−^ virions fails.

Whatever cell type MuHV-4 first targets, nasal host entry makes sense in its seclusion from any salivary route of virion exit. All viruses, being non-motile, must somehow combine efficient capture for host entry with efficient release for exit. Unlike the gut, the respiratory tract presents an anatomical dead end, necessitating bidirectional traffic. Epidemic respiratory viruses manage this by replicating to high titre, causing epithelial destruction, and stimulating sneezing and coughing. Herpesviruses, in contrast, rely on the chronic, asymptomatic shedding of low virion numbers. MuHV-4-infected cells show reduced heparan sulfate expression (de Lima *et al.*, 2004) and secrete gp70 to block local heparan sulfate rebinding (Gillet *et al.*, 2007), but these mechanisms are unlikely to operate beyond the millimetre (100-cell diameter) scale; host exit means travelling centimetres. Alphaherpesviruses have contrived cutaneous exit routes that are anatomically distinct from mucosal entry. Nasal entry and salivary exit could achieve the same for gammaherpesviruses. Such a scheme is perhaps less obvious for EBV and KSHV, because human noses are used less than those of rodents, but it begs to be explored.
